# The Association between Enterovirus 71 Infections and Meteorological Parameters in Taiwan

**DOI:** 10.1371/journal.pone.0046845

**Published:** 2012-10-05

**Authors:** Hsiao-Ling Chang, Chia-Pin Chio, Huey-Jen Su, Chung-Min Liao, Chuan-Yao Lin, Wen-Yi Shau, Yun-Chan Chi, Ya-Ting Cheng, Yuan-Lin Chou, Chung-Yi Li, Kwo-Liang Chen, Kow-Tong Chen

**Affiliations:** 1 Division of Surveillance, Center for Disease Control, Department of Health, Taipei, Taiwan; 2 School of Public Health, National Defense Medical Center, National Defense University, Taipei, Taiwan; 3 Department of Bioenvironmental System Engineering, National Taiwan University, Taipei, Taiwan; 4 Department of Environmental and Occupational Health, College of Medicine, National Cheng Kung University, Tainan, Taiwan; 5 Research Center for Environmental Changes, Academia Sinica, Taipei, Taiwan; 6 Division of Health Technology Assessment, Center for Drug Evaluation, Taipei, Taiwan; 7 Department of Statistics, National Cheng Kung University, Tainan, Taiwan; 8 Department of Public Health, College of Medicine, National Cheng Kung University, Tainan, Taiwan; 9 Department of Industrial Engineering and Management, China University of Science and Technology, Taipei, Taiwan; University of Florida, United States of America

## Abstract

**Background:**

Enterovirus 71 (EV71) infections are a significant cause of neurological disorder and death in children worldwide. Seasonal variations in EV71 infections have been recognized, but the mechanisms responsible for this phenomenon remain unknown. The purpose of this study was to examine the relationship between meteorological parameters and EV71 infection.

**Methods and Findings:**

We analyzed the number of EV71 infections and daily climate data collected in Taiwan between 1998 and 2008 and used Poisson regression analysis and case-crossover methodology to evaluate the association between weather variability and the incidence of EV71 infection. A total of 1,914 EV71-infected patients were reported between 1998 and 2008. The incidence of EV71 infections reflected significant summertime seasonality (for oscillation, p<0.001). The incidence of EV71 infections began to rise at temperatures above 13°C (r^2^ = 0.76, p<0.001); at temperatures higher than approximately 26°C (r^2^ = 0.94, p<0.05), the incidence began to decline, producing an inverted V-shaped relationship. The increase in the incidence with increasing relative humidity was positive and linear (r^2^ = 0.68, p<0.05). EV71 infection was most highly correlated with temperature and relative humidity in the period that likely preceded the infection.

**Conclusion:**

Our study provides quantitative evidence that the rate of EV71 infection increased significantly with increasing mean temperature and relative humidity in Taiwan.

## Introduction

Enterovirus 71 (EV71) is a member of the Enterovirus genus of Picornaviridae [1]. As of 2008, EV71 had been implicated in approximately 27 outbreaks in several regions worldwide since its discovery in 1969 in California; however, it was not until 1997 that the prevalence of EV71 infection increased significantly in Southeast Asia [2], [3]. Along with poliovirus, EV71 is an important cause of neurological disorders and deaths in children [4].

The impact of EV71 infection is greatest during the summer months in Asia [5]–[10], and epidemics recur with a seasonal pattern. Current hypotheses explaining the seasonal pattern of EV71 infection include host immune competence fluctuations mediated by seasonal factors, such as melatonin or vitamin D levels [11]; seasonal, behavioral factors unrelated to weather, such as school attendance and indoor crowding [12]; and environmental factors, including temperature [6]–[8] and relative humidity [6]–[8]. However, human behavioral factors alone do not appear to account for the seasonal pattern observed for certain cases of EV71 infection, including cases that do occur in school-aged children or in association with household crowding [13].

If temperature and humidity have a role in determining EV71 infection rates, changes in such variables could increase this disease’s burden in the future. An understanding of the EV71 seasonality could also enhance the accuracy of surveillance systems and improve our ability to predict epidemics and establish effective preventive measures [14]. Accordingly, the objective of this study was to investigate a possible relationship between weather and the incidence of EV71 infections in Taiwan.

## Methods

Taiwan has a population of approximately 22.7 million people spread over a land area of 36,188 km^2^ (population density: 627 people/km^2^). Taiwan is located at 23°4′ N and 121°0′ E and has a subtropical climate, with temperatures ranging from cool to hot and with relatively high humidity throughout the year.

Institutional review board approval for this study was obtained from the National Cheng Kung University Hospital, and informed consent was obtained from all patients or their parents.

### Case Data

The method for case collections has been described in previous study [5], [15], [16]. Briefly, laboratory-confirmed cases of EV71 infections reported to the Center for Disease Control, Taiwan (Taiwan CDC) between April 1, 1998 and December 31, 2008 were included in this study. Laboratory evidence of EV71 infection was defined as the isolation of EV71 from a throat swab, rectal swab, or stool sample, or a greater-than-four-fold rise or seroconversion with respect to the EV71-neutralizing antibody titer in paired sera samples [5], [15], [16]. Age, sex, area of residence, and date of symptom onset constituted the available case information. The date of symptom onset was used to calculate the case counts per calendar month.

### Meteorological Data

Daily temperature (maximum and minimum daily mean) and relative humidity data for the study period were provided by the Taiwan Central Weather Bureau (http://www.cwb.gov.tw). Being a relatively small island, the mean meteorological data value for each calendar week across the island was judged appropriate for the purposes of this study.

### Statistical Analysis

The annual incidence of severe EV71 infection cases was calculated by dividing the number of severe cases in children under 15 years of age by the number of children of the same age, as reported in the Taiwan census between 1998 and 2008. Seasonal and temporal trends in the occurrence of EV71 infection cases were evaluated using an approach similar to that described by Fisman et al. [17], with the construction of Poisson regression models that incorporated yearly terms:
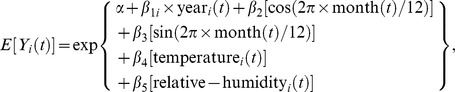


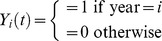


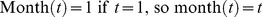
where *t = *1,2,….12, and Y*_i_*(*t*) denotes the number of cases at month *t* in year *i*, *α* is a constant, each *β* term denotes a regression coefficient for year or month, *t* indexes the month from January 1998 to December 2008, and *i* indexes the 10 years from 1998 to 2007. The indicator function year*_i_* (*t*) indicates whether is in year *i* (1 = yes, 0 = no). For example, year 1998 is represented by year_1_(*t*) = 1, year_2_(*t*) = 0, …, year_10_(*t*) = 0, and the reference year 2008 is represented by year*_i_*(*t*) = 0 for all *i*. The function month(*t*) maps to a month number, i.e., 1 to 12 for January to December. Temperature*_i_*(*t*) is temperature at month *t* in year *i*, similarly, relative humidity*_i_*(*t*) is relative humidity at month *t* in year *i*. The association between monthly case counts and meteorological exposures was explored through the construction of univariable and multivariable Poisson regression models. We also used a less restrictive approach to smoothing, using cubic splines to account for annual variations over the 11-year period. To avoid the pitfalls associated with both overfitting and underfitting, we used Akaike’s information criterion (AIC) to optimize the knots within the spline model [18]. Multivariable models were created using a backwards-elimination algorithm, with covariates retained for P≤0.2.

To explore the association between EV71 infection and different temperatures and relative humidity levels, we calculated the occurrence of EV71 infections at different temperatures and relative humidity levels [19]. Based on previous studies [6], [8], [20], [21], we hypothesized that changes in temperature and relative humidity would affect the survival and transmission of EV71 and therefore might influence the transmission activity among EV71-infected patients in a given population. The average occurrence of EV71 infection (*N_T_*) in different temperature domains (T to T+ΔT) was calculated using the following formula [19]:
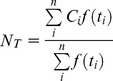



Here, *i* is a sequence from 0 to *n*, *t_i_* is the mean temperature for the *i*th 7-day period, *C_i_* is the total number of EV71 infection cases for the *i* +2th 7-day period, and *f*(*t_i_*) is a function with the following results:
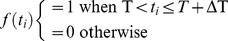



The numerator on the right side of the equation represents the sum of all *C_i_* comprising the 7-day mean temperatures (*t_i_*) within the temperature domain of T to T+ΔT during the data period. The denominator is the total number of occasions with T<*t_i_* ≤T+ΔT during the same data period.

Similarly, the average occurrence of EV71 infection (*N_H_*) in different relative humidity domains (H to H+ΔH) was determined using the following formula:
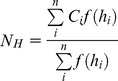



Here, *i* is an index from 0 to *n*, *h_i_* is the mean relative humidity for the *i*th 7-day period, *C_i_* is the total number of EV71 infection cases from the *i* +2th 7-day period, and *f*(*h_i_*) is a function with the following results:
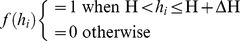



To further evaluate the association between meteorological variables and EV71 infection incidence, we used a case-crossover approach [22]. This study design is analogous to the case-control design but is characterized by self-matching, with individuals serving as their own controls [15], [18]. It was specifically developed to study the effect of transient exposures on the risk of acute events [22]. In case-crossover studies of environmental exposures, each subject’s exposure prior to a case-defining event is compared with his or her own exposure during a control period when he/she had not yet been diagnosed as a case. For each case, we selected one control date from the same day of the week four weeks before the case date [23], [24]. A case day was defined as the day on which the first symptom of the EV71 infection episode began and case period is 0–14 days prior to that day. Control day was chosen four weeks before the index event, and the control period is 0–14 days prior to the control day (28–42 days prior to the case day) to effectively control for temporal trends but without overlap between the case and control exposure periods. The analysis of case-crossover data is an application of standard methods for stratified data analysis. We performed conditional logistic regression, stratifying on each case, to obtain exposures odds ratios (ORs) as estimates of incidence rate ratios and 95% confidence intervals (CIs) associated with meteorologic variables.

Estimates of the plausible effect period were based on the incubation period of hand-foot-mouth disease (HFMD), which is usually 3 to 6 days but may range from 1 to 10 days [25]. The averaged daily measurements of environmental variables were used to determine exposures. Incidence rate ratios (IRRs) for occurrence of cases, based on weather effects, were approximated through the construction of conditional logistic regression models [26].

All analyses were performed using SAS Version 9.2 (SAS Institute Inc, Cary, NC, USA). The level of significance was set at p<0.05.

## Results

### Epidemiological Profile of EV71-infected Patients in Taiwan

Table S1 presents the number of cases and annualized rate according to sex, age, and region. During the study period, 1,914 cases of EV71 infection were reported to the Taiwan CDC. The annual incidence (case per 100,000) of reported EV71 infections was approximately 3.64 (range 0.25 to 8.76). EV71 infection was more common in males than in females (4.24 vs. 2.99). The annual incidence decreased as age increased, with a peak incidence rate (19.46) in 1-year-olds (p<0.001). The annual incidence was highest in the southern region (5.12) of Taiwan and lowest in the northern region (2.40) (p<0.001). Reported fatalities produced a case-fatality rate of 13.4% (95% confidence interval [CI] 11.9–15.0).

### Time Trends, Seasonality and Weather Effects

We created Poisson regression models to assess seasonality and time trends using monthly aggregate case counts. Using a model that incorporated terms for the calendar year, sine and cosine, we found strong evidence of a seasonal pattern of EV71 infection (for seasonal oscillation, p<0.001) (Figure S1). Case counts increased in the summer (June, July, August) (IRR = 4.02, 95% CI: 3.36–4.80), spring (IRR = 2.93, 95% CI: 2.43–3.52), and fall (IRR = 2.09, 95% CI: 1.72–2.53) relative to winter (December, January, February). Actual case counts and the number of cases fitting to data using Poisson regression are presented in Figure S1.

Table S2 presents data on meteorological factors and the incidence of EV71 infections. Univariable Poisson models identified numerous meteorological factors associated with the incidence of EV71 infections; however, when annual trends and oscillatory or cubic spline smoothers were incorporated into the models, the mean relative humidity and mean temperature were the only factors found to be independently associated with EV71 infection. No modification of the effect of temperature and humidity by age group or sex was detected.

### Temperature, Relative Humidity, and EV71 Infection Activity

The incidence of EV71 infection and temperature is shown in Figure S2 (A). The incidence of EV71 infection exhibited a different association with temperature. The risk of infection began to rise at 13°C (r^2^ = 0.76, p<0.001). When the temperature was higher than approximately 26°C, the risk of infection began to decline (r^2^ = 0.94 p<0.05), producing an inverted V-shaped relationship. The average case count (*N_T_*) increased by 28% (95% CI: 7–49%) for each 1°C increase in temperature ≤26°C; at temperatures >26°C, the average case count (*N_T_*) decreased by 30% (95% CI: 17–43%) for each 1°C increase.

The variation in average occurrence of EV71 infection in relation to different relative humidity domains (*N_H_*) is shown in Figure S2 (B). The pattern shows a positive slope with respect to relative humidity (r^2^ = 0.68, p<0.05). A 5-unit increase in humidity correlated with an 80% increase in the case count of EV71 infections (95% CI: 5–155%).

### Case-crossover Analysis of Acute Weather Effects

Figure S3 shows the odds ratios with respect to EV71 infection per degree increase in temperature and per unit (5%) increase in relative humidity during the same day and in the subsequent 14 days. Increased occurrence of EV71 infection was significantly associated with increased mean temperature and relative humidity in the period that preceded infection. Specifically, 1°C increase in mean daily temperature was associated with a statistically significant 26–39% higher incidence of reported EV71 infection (Figure S3). Similarly, relative humidity was significantly associated with the incidence of reported EV71 infection 5–14 days later (Figure S3).

## Discussion

EV71 is a growing public health concern in Asia [2], [3]. We analyzed data collected by public health authorities in Taiwan over an 11-year period, using both traditional methods for time series data and a case-crossover methodology. We found that EV71 infections occurred predominantly during the summer months but that infections were closely associated with humid periods during those months. This finding is consistent with the nature of EV71 species [1], [27]–[29]. Our findings also provide novel insights into the importance of the physical environment in determining the incidence and seasonality of EV71 infection in the developed world.

Enteroviruses that cause vesicular exanthema can presumably be spread by direct or indirect contact with vesicular fluid that contains infectious virus particles [1]. In addition, enteroviruses have been isolated in the highest titer and in stool specimens but can also be isolated from respiratory secretions [1]. Therefore, fecal-oral transmission and spread by contact with respiratory secretions are considered the most important transmission modes. It has been shown that the incidence of infectious diseases caused by gastrointestinal viruses, including rotavirus and poliovirus, is affected by seasonal changes in humidity. These changes have been credited with improving the duration of viral survival [27], [30] and virulence [29] in the environment, and such changes increase the number of opportunities for hosts to become inoculated as a result of contact with contaminated surfaces, aspiration of contaminated fomites, or inhalation of aerosols produced by the coughs of infected individuals [11], [31].

Our study found that the incidence of EV71 infections began to rise at temperatures above 13°C; at temperatures higher than approximately 26°C, the incidence began to decline, producing an inverted V-shaped relationship. We also found the morbidity due to EV71 infection increased during which the relative humidity is higher. Transmission of EV71, as with other enteroviruses, is assumed to be person to person, from feces or oropharyngeal secretions to the mouth, nose, or eyes, transferred via hands or formites [1]. However, empirical evidence to support this mode of transmission is limited to a few studies [12], [13], showing weak or no association with kindergarten attendance or household crowding [32]. Influenza causes significant morbidity in tropical regions; however, unlike in temperate regions, influenza in the tropics is not strongly associated with a particular season [33]. Lowen et al. [33] proposed that aerosol transmission plays a major role in influenza transmission in temperate climates, while contact or close-range spread are more important in the tropics. The effects of temperature and humidity on enterovirus transmission are not known. Although the Taiwanese government has implemented several prevention measures for EV71, including hand-washing and health education, EV71 infection epidemics have continued to occur annually since the EV71 outbreak in 1998 [5], [15]. Further studies are needed to determine whether our observation of a summer peak for EV71 infection is a result of different transmission routes.

This study found that male patients outnumbered female patient by 1.4∶1 (4.24 vs.2.99 per 100,000). This predominance has been observed in other enteroviral infections, in which the male to female ratio from 1.5∶1 to 2.5∶1 [34], [35]. These results cannot be reasonably explained at present, but they may suggest a susceptibility at the host genetic level [35].

Our study found that the incidence rate was highest in the southern region. The reasons for this are obscure. There were no known risk factors for severe of fatal infection, although many were considered. Severe cases occurred primarily in children who were less than 5 years old [5]. The case fatality rate was highest among children who were 7 to 12 months old [4].

There are still many unsolved questions about the annual fluctuations. Why did enteroviruses fairly prevalent cause epidemics in the years (1998, 2000, 2001, 2002, 2003, 2005, 2008), but there was very little disease in the years (1999, 2004, 2006, 2007)? It is postulated that the disease annual fluctuations can be caused by many factors, including host susceptibility, changing in contact rates between susceptible and infectious individuals, changing in pathogen transmission rates, and climate factors [36], [37]. This has been extensively studied as the susceptible/exposed/infectious/removed (SEIR) model [6], [38], but definite conclusions are not yet available.

The strength of this study was based on its analysis of laboratory-confirmed cases and, more importantly, clinically significant infections. The present study has several methodological limitations. First, many of the findings were based on surveillance data; therefore, a reporting bias may have affected the results. This bias can occur anywhere in the reporting chain, from a patient’s tendency to seek health care to the recording of the case in the disease registry. It is thought that many notifiable infectious diseases are under-reported [39]. Therefore, there may have been cases of EV71 infection in Taiwan during the study period that were not included in our analyses. However, this would have biased our result only if environmental effects (e.g. temporal heterogeneity due to increased burden in peak times) were somehow correlated with likelihood of disease reporting [18]. Second, we were not able to examine the effects of individual susceptibility; therefore, an investigation of the specific role of social and demographic factors in the spread of EV71 infections is critical for future studies. Third, the seasonal pattern of EV71 infection might result from unmeasured factors other than weather. These factors could include seasonal patterns linked to human activity and environmental factors other than weather.

Finally, there may be concerns about the lack of outpatient cases to examine all the effects of EV71 infection in the models. However, hospitalized patients with severe disease represented a small but important proportion of those infected and uniformly distributed throughout the island [4]. It seems that our results reflect the impact of the most common cause of EV71 infection.

In conclusion, the results of this study indicate that warmer temperature and elevated humidity would lead to an increased the rate of EV 71 infection in Taiwan. These results suggest that the preventive measures for controlling the spread of EV71 infection, particularly in younger children, should be considered during extended period of warmer temperature and elevated humidity.

## Supporting Information

Figure S1 **Trends in enterovirus 71 (EV71) infection cases in Taiwan.** The bars represent actual numbers, and the solid curve depicts the numbers of cases fitted to the data using Poisson regression model incorporating year, seasonal oscillatory term, temperature, and relative humidity; and the dashed curve depicts the number of cases fitted to the data using a model incorporating natural cubic splines, temperature terms, and relative humidity. The occurrence of cases was seasonal (with a summer predominance).
(TIF)

Figure S2 **The occurrence of enterovirus 71 (EV71) infection cases and variations in temperature.** The average EV71 infection occurrence (*N_T_*) was defined as the average number of EV71 infection cases observed during a 7-day period for a given temperature domain (A). The occurrence of EV71 infection cases and variations in relative humidity. The average occurrence of EV71 infection cases (*N_H_*) was defined as the average number of EV71 infection cases observed during a 7-day period for a given relative humidity domain (B).
(TIF)

Figure S3 **Conditional logistic regression results for enterovirus 71, with temperature and humidity as explanatory variables.** Separate analyses were conducted for each of 0–14 days prior to case occurrence, with the control date being 28 days prior to that date. Odds ratios (and 95% confidence intervals) represent the relative odds of disease for a 1 degree increase in temperature or a 5% rise in humidity.
(TIF)

Table S1 **Characteristics of children with EV71 infection in Taiwan from 1998 to 2008.**
(DOC)

Table S2 **Weekly weather patterns 8–14 days prior symptom onset and the incidence of EV71 infections in Taiwan, 1998–2008.**
(DOC)
