# Supplementary figures and images for: The Association between Enterovirus 71 Infections and Meteorological Parameters in Taiwan
Source: PLoS One. 2012 Oct 5;7(10):e46845. doi: 10.1371/journal.pone.0046845 (PMC3465260; doi:10.1371/journal.pone.0046845)

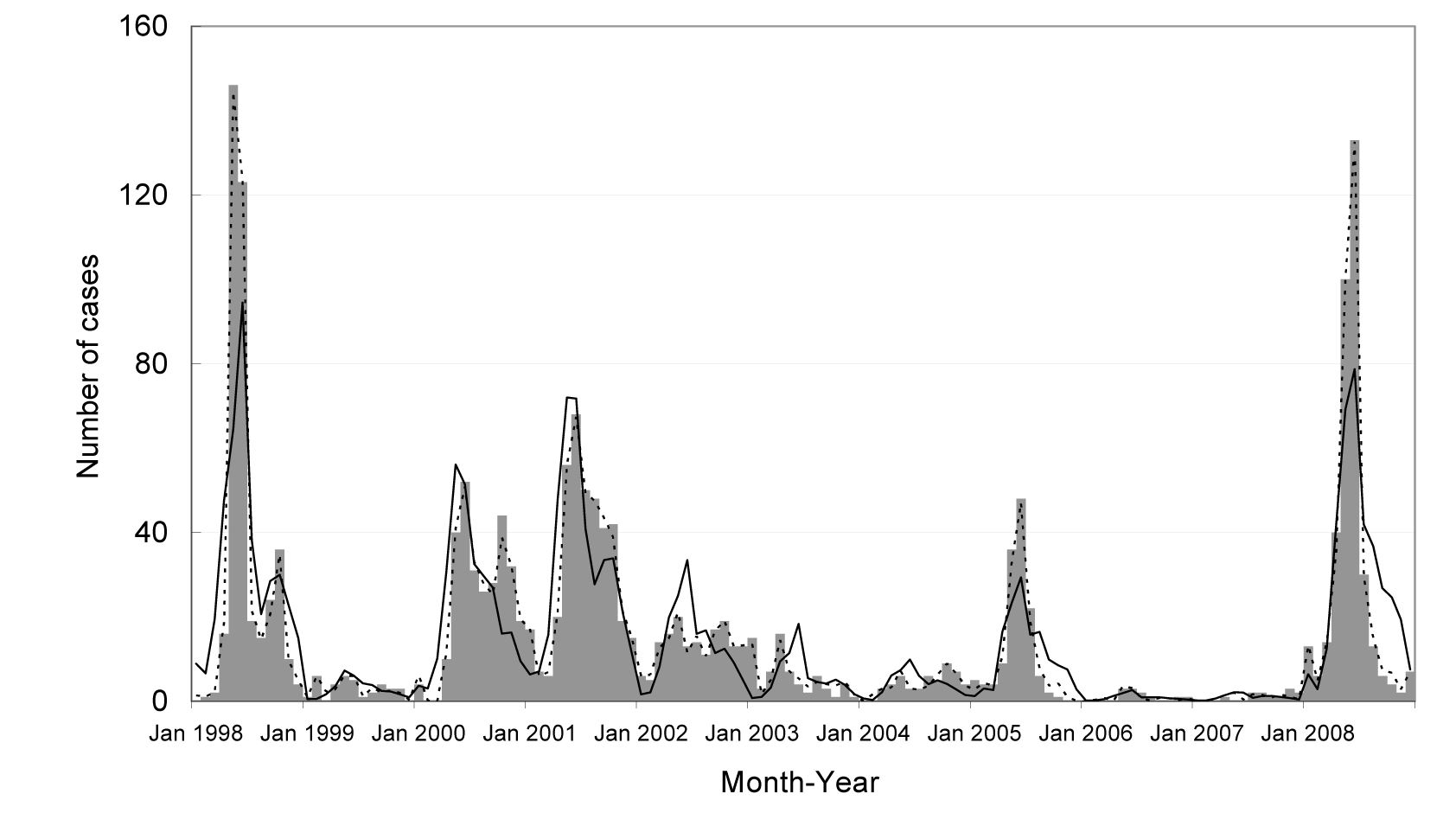

Supplement: Figure S1 — Trends in enterovirus 71 (EV71) infection cases in Taiwan. The bars represent actual numbers, and the solid curve depicts the numbers of cases fitted to the data using Poisson regression model incorporating year, seasonal oscillatory term, temperature, and relative humidity; and the dashed curve depicts the number of cases fitted to the data using a model incorporating natural cubic splines, temperature terms, and relative humidity. The occurrence of cases was seasonal (with a summer predominance). (TIF) [file pone.0046845.s001.tif]

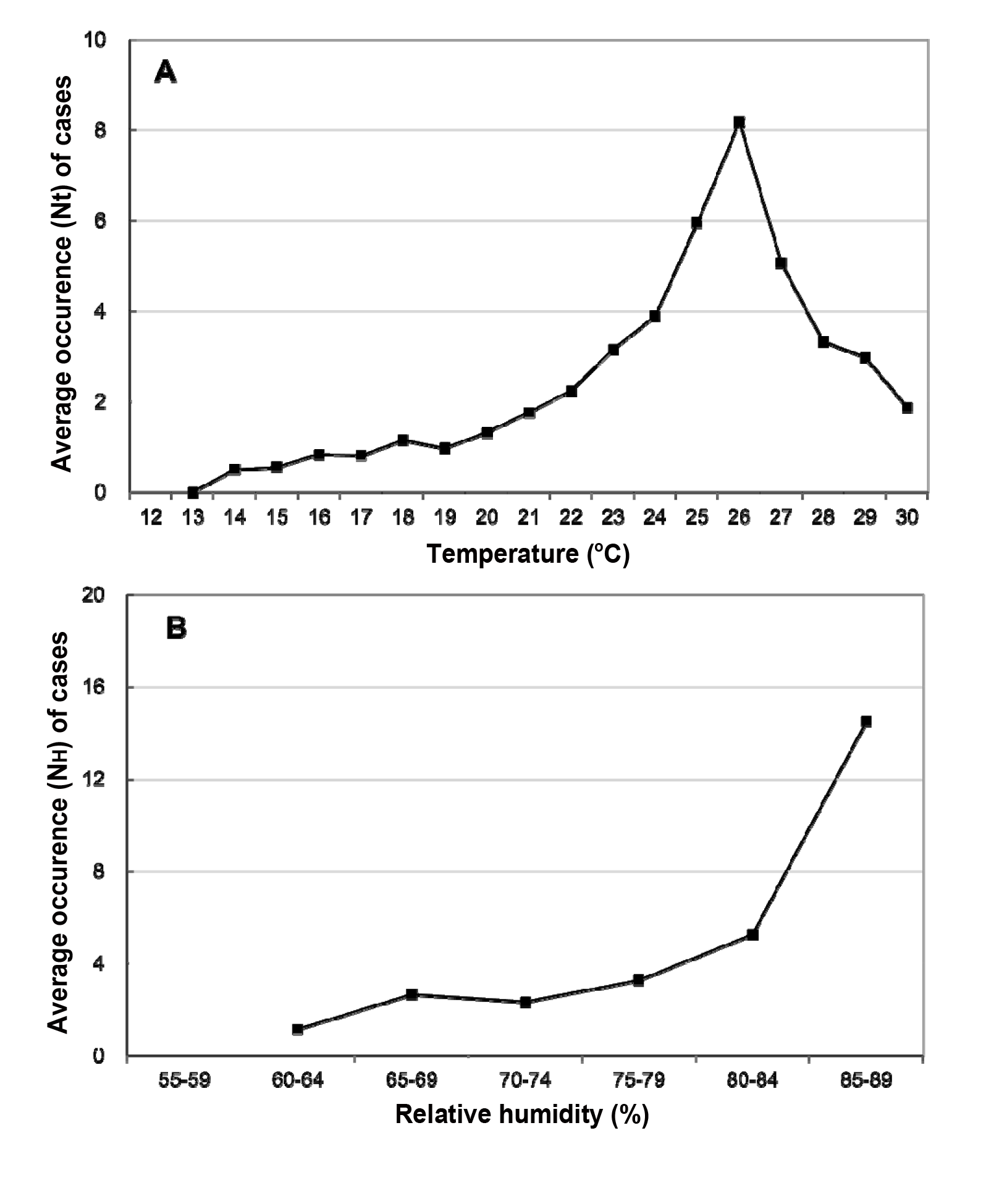

Supplement: Figure S2 — The occurrence of enterovirus 71 (EV71) infection cases and variations in temperature. The average EV71 infection occurrence (NT) was defined as the average number of EV71 infection cases observed during a 7-day period for a given temperature domain (A). The occurrence of EV71 infection cases and variations in relative humidity. The average occurrence of EV71 infection cases (NH) was defined as the average number of EV71 infection cases observed during a 7-day period for a given relative humidity domain (B). (TIF) [file pone.0046845.s002.tif]

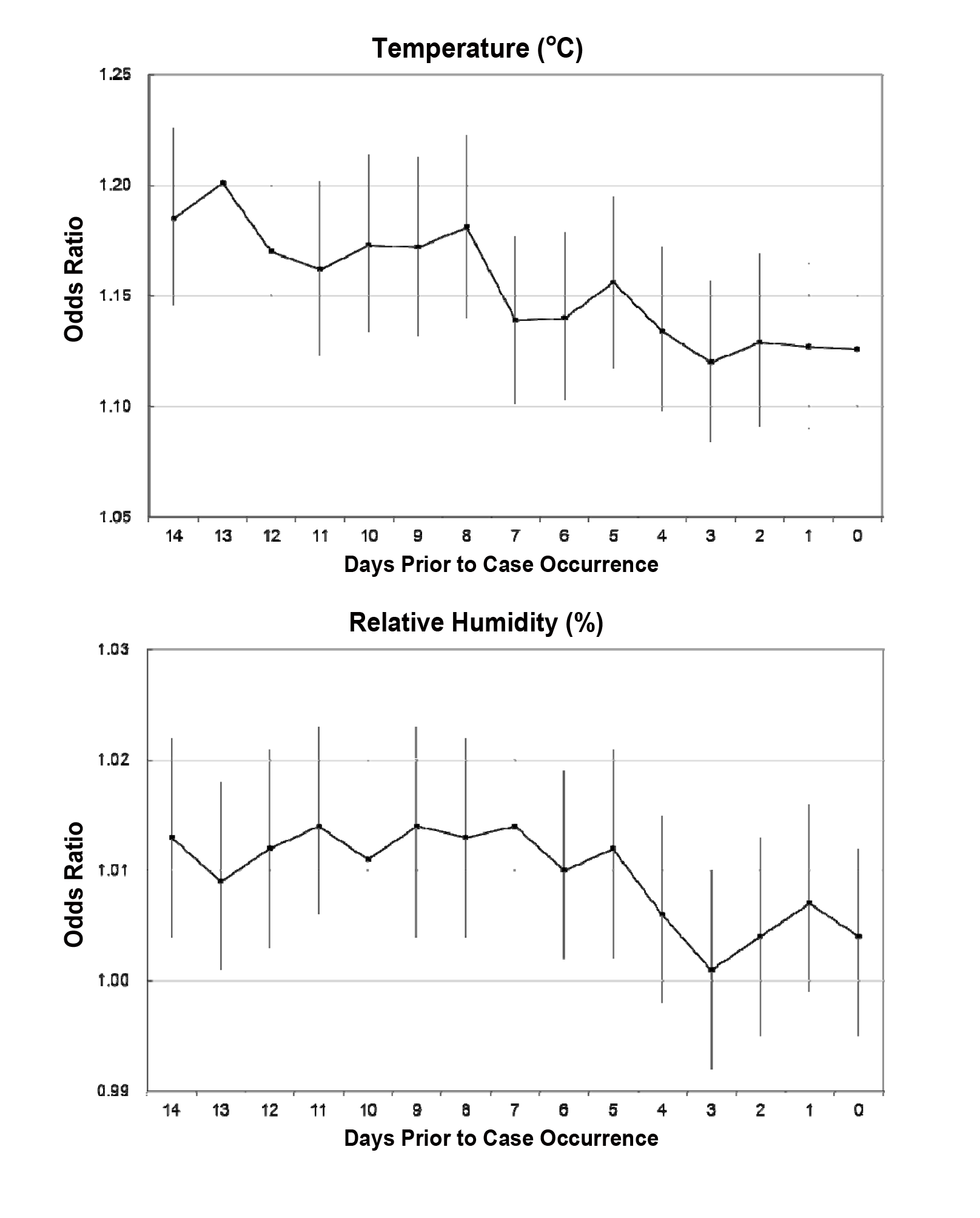

Supplement: Figure S3 — Conditional logistic regression results for enterovirus 71, with temperature and humidity as explanatory variables. Separate analyses were conducted for each of 0–14 days prior to case occurrence, with the control date being 28 days prior to that date. Odds ratios (and 95% confidence intervals) represent the relative odds of disease for a 1 degree increase in temperature or a 5% rise in humidity. (TIF) [file pone.0046845.s003.tif]
