# Supplementary material for: The Association between Enterovirus 71 Infections and Meteorological Parameters in Taiwan
Source: PLoS One. 2012 Oct 5;7(10):e46845. doi: 10.1371/journal.pone.0046845 (PMC3465260; doi:10.1371/journal.pone.0046845)
Supplement: Table S1 — Characteristics of children with EV71 infection in Taiwan from 1998 to 2008. (DOC) [file pone.0046845.s004.doc]

Table S1. Characteristics of children with EV71 infection in Taiwan from 1998 to 2008.

| Variables | Case (%) | Annual incidence rate per 100,000 |
| --- | --- | --- |
| Total | 1,914 (100) | 3.64 |
| Sex |  |  |
| Male | 1,159 (60.6) | 4.24 |
| Female | 755 (39.4) | 2.99 |
| Age (years) |  |  |
| < 1 | 495 (25.9) | 19.23 |
| 1 | 561 (29.3) | 19.46 |
| 2-3 | 569 (29.7) | 9.95 |
| 4-5 | 200 (10.5) | 3.18 |
| 6-15 | 89 (4.6) | 0.25 |
| Regions |  |  |
| Northern | 566 (29.6) | 2.40 |
| Central | 584 (30.5) | 4.26 |
| Southern | 718 (37.5) | 5.12 |
| Eastern | 46 (2.4) | 3.42 |
| Deaths | 256 (13.4) | --- |

EV71 = enterovirus 71.
