# Supplementary material for: The Association between Enterovirus 71 Infections and Meteorological Parameters in Taiwan
Source: PLoS One. 2012 Oct 5;7(10):e46845. doi: 10.1371/journal.pone.0046845 (PMC3465260; doi:10.1371/journal.pone.0046845)
Supplement: Table S2 — Weekly weather patterns 8–14 days prior symptom onset and the incidence of EV71 infections in Taiwan, 1998–2008. (DOC) [file pone.0046845.s005.doc]

Table S2. Weekly weather patterns 8-14 days prior symptom onset and the incidence of EV71 infections in Taiwan, 1998-2008

| Meteorological element | Univariable models | | | Multivariable Model Including Oscillatory Seasonal Smoothers and Annual Trend | | | Multivariable Model Including Cubic Splines | | |
| --- | --- | --- | --- | --- | --- | --- | --- | --- | --- |
|  | IRR | 95% CI | P value | IRR | 95% CI | P value | IRR | 95% CI | P value |
| Maximum temperature, mean, ℃ | 1.13 | 1.12, 1.15 | <0.001 |  |  |  |  |  |  |
| Minimum temperature, mean, ℃ | 1.13 | 1.12, 1.15 | <0.001 |  |  |  |  |  |  |
| Mean temperature, mean, ℃ | 1.13 | 1.12, 1.15 | <0.001 | 1.09 | 1.02, 1.16 | 0.009 | 1.16 | 1.14, 1.19 | <0.001 |
| Relative humidity, mean, ％ | 1.19 | 1.17, 1.21 | <0.001 | 1.08 | 1.05, 1.10 | <0.001 | 1.04 | 1.02, 1.06 | <0.001 |
| Atmospheric pressure, mean, kPa | 1.10 | 1.09, 1.11 | <0.001 |  |  |  |  |  |  |
| Precipitation, mm | 1.09 | 1.08, 1.10 | <0.001 |  |  |  |  |  |  |

Note: EV71 = enterovirus 71. Pressure and precipitation were excluded because of their collinearity with the mean temperature.
